# Ancient Case of Homicidal Insanity

**Published:** 1847-02

**Authors:** 


*Ancient case of Homicidal Insanity.* From the Connecticut Courant, of 1785. Litchfield, Conn. Nov. 15*th*, 1785.—"Last Wednesday Thomas Goss, late of Barkhamsted, was executed at this place, pursuant to the sentence of the Superior Court, for the murder of his wife. It seems he had adopted the idea sometime in October, 1784, that wizards and witches haunted him, and under pretence that his wife was a witch, he justified his conducted in depriving her of life. Under such infatuation, he ordered his attorney in most peremptory language, not to apply for a reprieve to any human tribunal, alleging that his Heavenly Father had forbidden all such proceedings. He called himself the second Lamb of God: said he was brother of Jesus Christ; and sometimes said he was the child born of the woman mentioned in the Revelation of St. John, before whom the dragon stood ready to devour the child, &c. To such extravagant ideas he added that the sheriff could not hang him; that his Heavenly Father would interpose, if the attempt were made, and he be liberated; and that thirty thousand males above fifteen years of age, would be instantly killed by the shock, in North America. He pertinaciously adhered to such opinions to the last moment of his life. The night preceding his execution, he slept well. In the forenoon of the same day, he slept calmly for a considerable length of time:—at dinner, ate heartily. On his way to the gallows, and while there, he appeared calm and unmoved; not the least emotion could be discerned in his countenance, nor the least perturbation in his speech, and the last word he said was, that the sheriff could not hang him."

A distinguished gentleman of Connecticut who was present at the trial and execution informed Dr. Brigham, that Goss, when on the gallows, exhibited the utmost unconcern; leisurely took a chew of tobacco, and that this, and his indifference, so exasperated the people assembled, that they rejoiced in the death of one they believed hardened in guilt,—one whom we must now regard as insane, and whose life, had the circumstances occurred at the present day, would not we believe have been thus sacrificed.

Goss after killing his wife immediately informed the neighbors, and claimed that he had acted in obedience to the command of Scriture, "Thou shalt not suffer a witch to live."—*Amer. Jour. of Insanity.*

